# The impact of post-stroke fatigue on work and other everyday life activities for the working age population – a registry-based cohort study

**DOI:** 10.1080/07853890.2023.2269961

**Published:** 2023-10-18

**Authors:** Jessica Vollertsen, Mathilda Björk, Anna-Karin Norlin, Elin Ekbladh

**Affiliations:** a Department of Rehabilitation, and Department of Health, Medicine and Caring Sciences, Linköping University, Motala, Sweden; b Pain and Rehabilitation Center, and Department of Health, Medicine and Caring Sciences, Linköping University, Linköping, Sweden; c Department of Health, Medicine and Caring Sciences, Linköping University, Linköping, Sweden

**Keywords:** Post-stroke fatigue, everyday life, return to work, registry study, ADL, complex activities, rehabilitation, life after stroke

## Abstract

**Introduction:**

Life after stroke is a comprehensive area that involves engagement in meaningful everyday activities, including work, and can be adversely affected by post-stroke fatigue. This study investigates post-stroke fatigue, its development over time, and its impact on return to work and other everyday life activities. In addition, we investigated whether post-stroke fatigue could predict functioning in everyday life activities one year after stroke.

**Material and methods:**

This prospective registry-based study includes 2850 working age (18 – 63 years) patients registered in the Swedish Stroke Register (Riksstroke) during year 2017 and 2018. Post-stroke fatigue and everyday activities were analyzed 3- and 12-months post-stroke.

**Results:**

The mean age of the included participants was 54 years and the majority, 65%, were men. Three months post-stroke, 43% self-reported fatigue, at 12-months the proportion increased to 48%. About 90% of the patients were independent in basic ADL at 3-month. Dependence in complex activities one year post-stroke was significantly associated with fatigue. Not experiencing fatigue one year after stroke could predict positive functioning in everyday activities, increasing the chance of returning to work (OR = 3.7) and pre-stroke life and everyday activities (OR = 5.7).

**Conclusion:**

Post-stroke fatigue is a common persistent disability that negatively impacts complex activities; therefore, fatigue needs to be acknowledged and addressed long term after discharge.

## KEY MESSAGES

- People of working age who experience post-stroke fatigue encounter difficulties with complex rather than basic activities.
- Post-stroke fatigue may be developed when reclaiming complex activities; thus, long term routine assessments after discharge are recommended.
- Interventions addressing post-stroke fatigue are warranted, as the absence of post-stroke fatigue increases the chance of returning to everyday life, including work.

## Introduction

When planning rehabilitation to promote health after a stroke, it is crucial to consider the individual’s ability to function in various activities of daily life [1]. Prior research has shown a growing incidence of stroke in the working age population [2,3], with one in six stroke patients in Sweden aged between 20 and 64 years [4]. Returning to work after a stroke is a significant objective of rehabilitation for many working age individuals [5], as it can enhance their health, autonomy, quality of life, and perceived participation [6,7]. Nevertheless, attempting to return to work while coping with post-stroke limitations can cause frustration, fear, and anxiety [8,9]. To achieve a sustainable work situation, it is essential to manage different areas of daily life as a cohesive whole, such as family life, household duties, and work responsibilities [10,11]. A lack of practical support in everyday life, such as childcare and household chores, can pose a barrier to returning to work [10]. Even after attaining a high level of physical recovery, individuals who have suffered a stroke often encounter difficulties in reintegrating into the workforce and maintaining employment [12,13]. From a societal perspective, return to work is economically advantageous as the indirect societal costs associated with productivity loss are immense. For people between 18 and 63 years old, the average indirect costs during the first year after the onset of stroke amount to about 17400 euros per person and in the second year 12200 euros per person [14].

A symptom that has been identified as a crucial barrier to returning to work for both men and women is post-stroke fatigue (PSF) [15,16]. For people returning to work within the first year after stroke, PSF has been rated as the greatest impairment barrier [5]. Fatigue can be defined as disproportionate mental or physical fatigue and lack of energy triggered by simple activities, where fatigue does not improve with normal rest [17]. Due to the large heterogeneity in studies regarding time and type of assessment, stroke diagnosis, and where the studies were conducted, the prevalence of PSF varies between 42% and 62% [18]. However, the underlying mechanisms of PSF remain unknown. The experience of PSF in the recovery process at different time points can be explained by several factors. The early onset of PSF could be explained by biological factors such as stroke severity; later, in the recovery process, PSF could be associated with psychological and behavioral factors [19]. A differential diagnosis of PSF is depression, and previous studies have demonstrated correlations between depression and PSF [19–21]. Although PSF and depression are related, they should be considered as two separate sequelae of stroke [22]. Female sex has been found to be associated with PSF [21,23]. Evidence is inconclusive regarding the correlation between age and PSF, as both young and old age have been found to be possible predictors of PSF [17].

Post-stroke fatigue has been identified as one of the most challenging impairments to manage in everyday life following a stroke according to qualitative studies [8,24]. Moreover, individuals under the age of 70 years have ranked PSF as the most important research area related to life after stroke [25]. Rushing back to work too early or extending work hours too quickly after stroke may lead to increased PSF [26]. Working age individuals normally engage in complex activities as part of their everyday lives, including work, family life, childcare, and transportation, which necessitate planning, multitasking, and problem-solving skills. Still, many people return to work and at the same time trying to regain meaningful activities in everyday life after stroke. However, few studies have focused on the role of work as an integral part of everyday life post-stroke. Therefore, it is essential to investigate the temporal aspect of PSF and its relationship with everyday activities, including work. In addition, there is a need to expand the existing knowledge base on PSF and everyday life using larger ­sample sizes.

This study investigates post-stroke fatigue, its development over time, and its impact on return to work and other everyday life activities. In addition, we investigated whether post-stroke fatigue could predict functioning in everyday life activities one year after stroke.

## Materials and methods

Data retrieved from the Swedish Stroke Register (Riksstroke) were used in this registry-based prospective cohort study. The registry covers all hospitals in Sweden admitting acute stroke patients. For each patient, data were collected by hospital personnel during the patient’s stay and through self-rated questionnaires 3 and 12 months after stroke onset [27]. Details are available at http://www.riksstroke.org/eng/.

Strengthening the Reporting of Observational Studies in Epidemiology (STROBE) Statement: Guidelines for reporting observational studies [28] were followed when applicable.

### Study population

All patients who were diagnosed with ischemic and hemorrhagic strokes (ICD-10 codes I61, I63, and I64) in 2017 and 2018 and were of working age at the time of stroke were eligible for inclusion in the study. Working age was defined as an age between 18 and 63 years. Because 65 years is the common retirement age in Sweden, the upper age limit was set to 63 years. Exclusion criteria were as follows: participants not being employed at the time of the stroke and/or not having responded to both the 3- and 12-month follow-up questionnaire.

### Variables

Demographic data concerning sex, age, diagnosis, hospital length of stay, activities of daily living (ADL), and living conditions at stroke onset were retrieved from data collected by personnel during the hospital stay. Self-rated feelings of depressed mood were retrieved from the 3- and 12-month follow-up questionnaires. The question was formulated as follows: Do you feel depressed? If you feel depressed, this question applies regardless of the reason for the depression?’ The response options were ‘Never or almost never’, ‘Sometimes’, ‘Often’, ‘Constantly’, and ‘Don’t know’. The variable was dichotomized. ‘Never or almost never’ and ‘Sometimes’ were defined as ‘No, depressed mood’ and ‘Often’ and ‘Constantly’ were defined as ‘Yes, depressed mood’. The response option ‘Don’t know’ was considered an internal drop-out, which resulted in 50 internal drop-outs at 3-months and 21 at 12-months.

### Post-stroke fatigue

Post-stroke fatigue was measured by a self-rated general experience of fatigue at 3- and 12-months after stroke. In Riksstroke, the question was formulated as follows:’ Do you feel tired? If you are tired, this question applies regardless of the reason for the tiredness’. The response options were’ Never or almost never’, ‘Sometimes’, ‘Often’, ‘Constantly’, and ‘Don’t know’. In this study, the PSF variable was dichotomized. ‘Never or almost never’ and ‘Sometimes’ were defined as ‘Not having PSF’, and ‘Often’ and ‘Constantly’ were defined as ‘Having PSF’. The response option ‘Don’t know’ was considered an internal drop-out, which resulted in 19 internal drop-outs at 3-months and 7 at 12-months.

### Everyday life activities

Everyday life activities were defined using six questions from the Riksstroke. Three questions were used to measure basic ADL (mobility, toilet visits, and dressing), and three questions were used to measure complex ADL (care of household, return to work, and returning to life and activities performed pre-stroke). Data were collected at 3- and 12-months for all variables except for return to work, which was only collected at 12-months.

The ability to move indoors and outdoors was measured using a three-point rating scale. The response options were ‘I can move both indoors and outdoors without the help of another person’, ‘I can move independent indoors, but not outdoors, without the help of another person’ and ‘I need help from another person when moving both indoors and outdoors’. The variable were dichotomized. Being able to move indoors and outdoors without help was defined as independent. If help was required, it was defined as dependent. The variables toilet visits, dressing, and care of household were self-rated as independent or dependent. The variable care of household had two additional response options: ‘Not relevant’ (3-months, *n* = 69; 12-months, *n* = 62) and ‘Don’t know’ (3-months, *n* = 28; 12-months, *n* = 29), which were considered as internal drop-outs.

Return to work was measured using a five-point rating scale referring to amount of time spent back at work compared to pre-stroke. The response options were ‘ Returned to work to the same extent as before the onset of stroke, ‘Returned to work to a lesser extent as before the onset of stroke,’ Not returned to work but planning to return, ‘No, cannot return to work’, and ‘Don’t know’. The variable was dichotomized with the response option ‘Returned to work to the same extent as before the onset of stroke’, defined as ‘Returned to work’, and the remaining response options together were defined as ‘Not returned to work’.

The ability to return to life and perform activities as before stroke onset was measured using a three-point scale. The response option ‘Yes’ was defined as ‘Returned to everyday life activities’. The two remaining response options ‘Yes, but not quite as before’ and ‘No’ were defined as ‘Not returned to everyday life activities’. The response option ‘Don’t know’ was considered an internal drop-out (3-months, *n* = 28; 12-months, *n* = 18).

The proportion of missing data varied between 8 and 9% for all variables collected at 3-months and between 0.5 and 1% for all variables collected at 12-months.

### Statistical analysis

Number, percentage, mean, and standard deviation (SD) were used to describe participants’ characteristics and functioning in everyday life. For comparison between groups i.e. patients reporting having PSF or reporting not having PSF, the Chi^2^ test (nominal variables), the Mann-Whitney U test (ordinal variables) and the Student’s *t*-test (interval variables) were used [29].

Four groups were created to describe the development of PSF between 3- and 12-months. The first group, ‘Absence of PSF’, consisted of those who were ‘Not having PSF’ at either 3- or 12-months. The second group, ‘Recovered PSF’, consisted of those who at 3-months were ‘Having PSF’ and improved and were ‘Not having PSF’ at 12-months. The third group, ‘Persistent PSF’, consisted of those who were ‘Having PSF’ at both 3- and 12-months. The fourth group, ‘Late onset of PSF’, consisted of those who were ‘Not having PSF’ at 3- months and ‘Having PSF’ at 12-months. The Chi^2^ test was used to analyze the development of PSF and its impact on functioning in everyday life activities. The analysis was conducted using dichotomized variables defining everyday activities 12-months after stroke onset. The Bonferroni test was used for post-hoc corrections [29].

Binary logistic regression was used to investigate whether PSF could predict functioning in everyday life activities one year after stroke. Separate regression models were created for each dichotomized variable that defined everyday life activities. Post-stroke fatigue was set as the independent variable and tested and adjusted for age, sex, and depressed mood as possible confounders. If the regression coefficient of the PSF changed by more than 15%, the added variable was considered a confounder [30].

The IBM Statistical Package for the Social Sciences version 28 [31] was used for all statistical analyses. All statistical tests were two-sided, with a significance level of 5%.

### Ethical approval and informed consent

This study was approved by the Swedish Ethical Review Authority (Dnr: 2020-01200). When admitted to the hospital, the participants were informed that the collected data in Riksstroke could be used for research and that they could withdraw their participation in the registry at any time. Furthermore, the participants were informed that answering the follow-up questionnaire was voluntary. According to the Swedish Data Protection Authority, the handling of data generated for quality registries represents an exception to the general rule, which requires written informed consent from patients. For these reasons, no written or oral informed consent was obtained from the participants.

## Results

**Figure 1.**
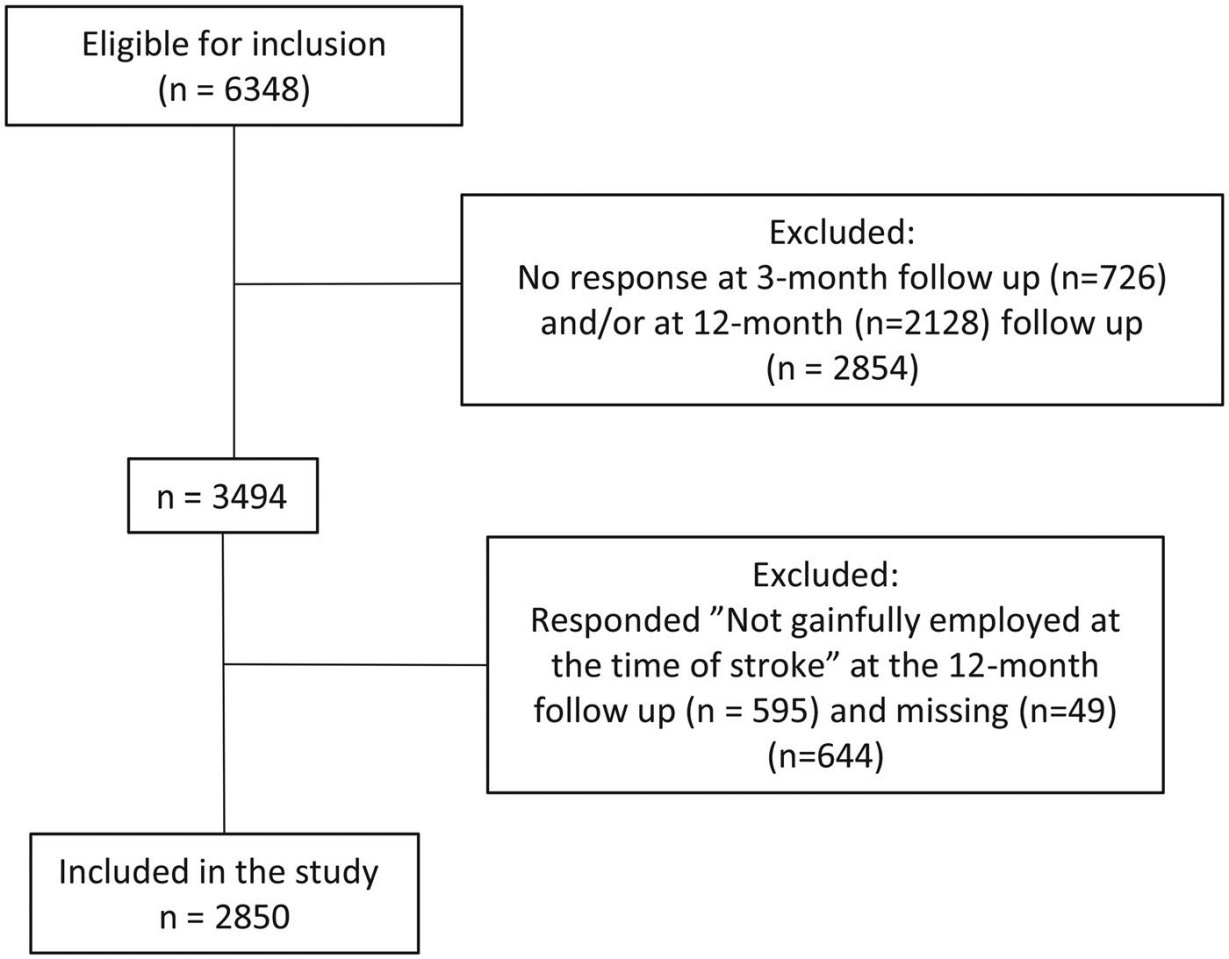
Flowchart of the study population.

A total of 6 348 individuals were eligible for the study. As 2 854 did not answer the 3-month and/or 12-month follow-up, they were excluded from the study. In addition, 644 individuals were excluded because they were not employed at the time of stroke or had not answered (*n* = 49). This resulted in a total of 2850 participants being included in the study (Figure 1). There was no significant difference in age between the included and excluded participants; however, there were significantly more women among the included participants than among the excluded participants (*X*^2^ = 4.366, df = 1, *p* = 0.037).

### Participants

Table 1 lists the characteristics and functioning in everyday life of the 2850 included participants. Approximately 65% of the participants were male. The majority of patients had a stroke onset between 55 and 63 years. Men were significantly older (*p* < 0.001) than women with a mean age of 54.5 years (SD 7.8) compared to women with a mean age of 52.6 years (SD 9.4). The most common diagnosis for both sexes was ‘Ischemic stroke’ (ICD-10 code I63) (86%), and nine participants were diagnosed with ‘Stroke, not specified as hemorrhage or infarction’ (ICD-10 code I64). The average length of hospital stay was 8.6 days. At hospital admission, 99% lived an independent life in their own home without home care and were independently able to perform basic ADL (toilet visit, dressing, and moving indoors and outdoors without help). The majority (75%) shared their household with a spouse, cohabitant, or another person.

**Table 1.** Respondents’ characteristics and differences in functioning in everyday activities in relation to experiencing or not experiencing post-stroke fatigue at 3- and 12-months.

	3 months				12 months			
	Total	Having PSF	Not having PSF	*p*	Total	Having PSF	Not having PSF	*p*
Total, *n*	2850	1107 (43)	1470 (57)			1345 (48)	1478 (52)	
Sex, *n* (%)								
Men	1846 (65)	602 (36)	1051 (64)	<0.001^a^		765 (42)	1065 (58)	<0.001^a^
Women	1004 (35)	505 (55)	419 (45)			580 (58)	413 (42)	
Age onset of stroke, (years) (mean + SD)	53.8 + 8.5	53.2 + 8.8	54.4 + 8.1	<0.001^b^		53.3 + 8.8	54.3 + 8.1	0.003^b^
Men	54.5 + 7.8	54.2 + 8.0	54.8 + 7.6	0.149^b^		54.2 + 8.2	54.7 + 7.5	0.189^b^
Women	52.6 + 9.4	51.9 + 9.4	53.5 + 9.1	0.007^b^		52.1 + 9.4	53.1 + 9.3	0.117^b^
Diagnosis, *n* (%)								
Ischemic stroke	2465 (86)	959 (87)	1288 (88)	0.598^a^		1159 (87)	1281 (87)	0.821^a^
Cerebral hemorrhage	376 (13)	142 (13)	179 (12)			180 (13)	194 (13)	
Hospital length of stay, (days per patient) (mean + SD)	8.6 + 13	8.9 + 13.3	7.4 + 10.7	<0.001^b^		9.6 + 14.5	7.7 + 11.3	<0.001^b^
Mobility, n (%)								
Independent	2352 (92)	967 (88)	1385 (95)	<0.001^a^	2596 (93)	1180 (89)	1416 (97)	<0.001^a^
Dependent	213 (8)	136 (12)	77 (5)		200 (7)	151 (11)	49 (3)	
Toilet visits, *n* (%)								
Independent	2469 (96)	1035 (94)	1434 (98)	<0.001^a^	2719 (97)	1269 (95)	1450 (99)	<0.001^a^
Dependent	101 (4)	69 (6)	32 (2)		85 (3)	67 (5)	18 (1)	
Dressing, *n* (%)								
Independent	2474 (94)	1018 (92)	1418 (97)	<0.001^a^	2676 (95)	1239 (93)	1437 (98)	<0.001^a^
Dependent	149 (6)	85 (8)	51 (3)		132 (5)	98 (7)	34 (2)	
Care of household, *n* (%)								
Independent	1990 (79)	722 (69)	1243 (88)	<0.001^a^	2248 (82)	926 (73)	1305 (91)	<0.001^a^
Dependent	521 (21)	329 (31)	175 (12)		487 (18)	350 (27)	131 (9)	
Return to work, n (%)								
Yes, same					1279 (45)	362 (27)	908 (61)	<0.001^c^
Yes, less					685 (24)	423 (31)	260 (18)	
No					886 (31)	560 (42)	310 (21)	
Returned to life and activities, *n* (%)								
Yes	788 (31)	133 (12)	645 (44)	<0.001^c^	987 (35)	189 (14)	790 (54)	<0.001^c^
Yes, but not quite as before	962 (37)	433 (40)	520 (36)		1175 (42)	662 (50)	504 (34)	
No	835 (32)	528 (48)	285 (20)		653 (23)	477 (36)	168 (12)	
Depressed mood, *n* (%)								
Yes, depressed mood	316 (12)	263 (24)	50 (3)	<0.001^a^	454 (16)	387 (29)	58 (4)	<0.001^a^
No, depressed mood	2231 (88)	825 (76)	1384 (97)		2350 (84)	935 (71)	1401 (96)	

*Note:*

^a)^ *Chi^2^ was applied, α = 0.05*.

^b)^ *Independent t-test (two-sided p) was applied, α = 0.05*.

c) *The Mann Whitney U was applied, α = 0.05.*

### Post-stroke fatigue and participants characteristics

At the 3-month follow-up, 43% of the participants reported that they experienced PSF; at the 12-month follow-up, the proportion had increased to 48%. On both occasions, significantly more women than men reported having PSF (3-months: *X*^2^ = 80.428, df = 1, *p* < 0.001; 12-months: *X*^2^ = 71.158 df = 1, *p* < 0.001) (Table 1). On average, participants who reported having PSF 3-months post-stroke were hospitalized for 8.9 days, and these hospitalizations were significantly longer than for those who reported not having PSF (7.4 days) (*p* < 0.001). Participants who reported having PSF at the 12-month follow-up, were hospitalized significantly longer (9.6 days) compared to those who reported not having PSF (7.7 days) (*p* < 0.001). Twenty four percent of the participants who reported having PSF at the 3-month follow-up stated that they had a depressed mood, compared to those who did not report having PSF (3%) (*X*^2^ = 243.5, df = 1, *p* < 0.001). One year after stroke, significant differences persisted: 29% of participants who reported having PSF had a depressed mood, compared to those who did not report having PSF (4%) (*X*^2^ = 330.3 df = 1, *p* < 0.001).

### Post-stroke fatigue and everyday activities

The majority of the participants were independent in mobility, toilet visits and dressing at the 3-months follow-up regardless of whether they reported having PSF or not. Participants who reported having PSF 3-month post-stroke were significantly more dependent in mobility (12%), toilet visits (6%) and dressing (8%) compared to those who reported not having PSF (mobility 5%, toilet visits 2% and dressing 3%) (*p* < 0.001) (Table 1). One year after stroke recovery, participants who reported having PSF remained significantly more dependent in mobility (11%), toilet visits (5%) and dressing (7%) compared to those who reported not having PSF (mobility 3%, toilet visits 1% and dressing 2%) (*p* < 0.001). Regarding more complex activities, participants who reported having PSF needed help taking care of the household to a significantly greater extent at both 3- (31%) and 12-months (27%) than those who did report not having PSF at 3- (12%) and 12-months (9%) (3-months: *X*^2^ = 133.6, df = 1, *p* < 0.001; 12-months: *X*^2^ = 155.2, df = 1, *p* < 0.001). Concerning return to work one year post-stroke, participants who reported having PSF 12 months after stroke onset had not returned to work to the same extent (27%) as those who reported not having PSF one year post-stroke (61%) (*Z* = −17.4, *p* < 0.001) (Table 1). When considering the ability to return to life and perform activities as before stroke onset: 3-months post-stroke 88% of the participants who reported having PSF had not been able to return to everyday life and activities to the same extent as before the stroke compared to 56% of the participants who reported not having PSF (3-months: *Z*= −19.2, *p* < 0.001). One year after stroke, 86% of the participants who reported having PSF had not returned to everyday life and activities compared to 46% of the participants who reported not having PSF (12-months: *Z* = −22.7, *p* < 0.001) (Table 1).

### Post-stroke fatigue and its development over time

When the development of the participants’ self-reported PSF was analyzed, 33% reported that they had PSF at both 3- and 12-months (Persistent PSF), and 14% reported a development of PSF between 3- and 12-months (Late onset of PSF) (Table 2). Significantly more women (44%) than men (27%) reported having PSF at both 3- and 12-months (*X*^2^ = 97.870, df = 3, *p <* 0.001).

**Table 2.** Differences in age and sex between the four groups created to describe the development of PSF between 3- and 12-months

	Absence of PSF (No PSF at 3 or 12 months)	Recovered PSF (PSF at 3 months and no PSF at 12 months)	Late onset of PSF (No PSF at 3 months and PSF at 12 months)	Persistent PSF (PSF at 3 and 12 months)	*p*
Total, *n* (%)	1100 (43)	252 (10)	361 (14)	841 (33)	
Age, mean + SD	54.7 + 7.8	52.8 + 8.5	53.5 + 8.7	53.2 + 8.8	<0.001^a^
Age, min – max	18–63	21–63	22–63	19–63	
Women, *n* (%)	291 (32)	92 (10)	126 (14)	405 (44)	<0.001^b^
Men, *n* (%)	809 (49)	160 (10)	235 (14)	436 (27)	

*Note:*

^a)^ *One way Anova Test was applied, α = 0.05*.

^b)^ *Chi^2^ was applied, α = 0.05*.

### Impact of the development of post-stroke fatigue on everyday life activities

The analysis revealed statistically significant differences between the group ‘Absence of PSF’ and the group ‘Persistent PSF’ regarding dependence in both basic and complex activities. Furthermore, there were statistically significant differences for all group comparisons, except when comparing ‘Recovered PSF’ with ‘Late onset of PFS’ regarding dependence in complex activities. For dependence in basic ADL, there were significant differences when comparing the ‘Absence of PSF’ group with another group. The direction of the differences indicated that participants who belonged to the ‘Persistent PSF’ group or the ‘Late onset of PSF’ group were more dependent in everyday activities (Table 3).

**Table 3.** Comparison between the different groups describing the development of PSF between 3- and 12-months regarding dependence in everyday life activities at 12-months.

	Dependent on help with mobility %	p	Dependent on help with toilet visits %	p	Dependent on help with dressing %	p	Dependent on help with care of household %	p	Not returned to work %	p	Not returned to life/activities %	p
Absence of PSF	2	<0.01	0.5	0.01	1	0.10	7	0.04	33	<0.01	42	<0.01
Recovered PSF	6		2		4		12		55		61	
Absence of PSF	2	<0.01	0.5	<0.01	1	0.02	7	<0.01	33	<0.01	42	<0.01
Late onset of PSF	9		3		4		20		63		80	
Absence of PSF	2	<0.01	0.5	<0.01	1	<0.01	7	<0.01	33	<0.01	42	<0.01
Persistent PSF	11		5		7		29		77		88	
Recovered PSF	6	0.74	2	5.66	4	5.06	12	0.11	55	0.39	61	<0.01
Late onset of PSF	9		3		4		20		63		80	
Recovered PSF	6	0.08	2	0.70	4	0.29	12	<0.01	55	<0.01	61	<0.01
Persistent PSF	11		5		7		29		77		88	
Late onset of PSF	9	1.93	3	0.47	4	0.23	20	<0.01	63	<0.01	80	<0.01
Persistent PSF	11		5		7		29		77		88	

Note: Chi^2^ was applied when comparing the groups, α = 0.05. Significance values has been adjusted by the Bonferroni correction for multiple tests.

### Prediction of functioning in everyday life activities one year after stroke

Logistic regression revealed that not having PSF 12-months after the onset of stroke was statistically significantly related to positive functioning in everyday life regarding both basic and complex activities (Table 4). Neither sex nor age had any influence on the chance of positive functioning in everyday life activities; however, depressed mood at 12-months was significantly related to PSF for all variables. The participants who reported not having PSF at the 12-month follow-up were almost six times more likely to self-report that they had been able to return to their life and everyday activities before stroke (OR = 5.7, *p* < 0.001). They were also almost four times more likely to self-report that they had been able to return to work to the same extent as before stroke (OR = 3.7, *p* < 0.001). Furthermore, they were almost three times more likely to be able to independently manage household chores (OR = 2.9, *p* < 0.001) (Table 4).

**Table 4.** Lack of post-stroke fatigue and prediction of positive functioning in everyday life activities one year after stroke.

Independence in activities	OR	Adjusted OR	95 % CI for adjusted OR	*p*
Mobility	3.698	2.359	1.639−3.394	<0.001
Dressing	3.343	1.896	1.212−2.968	0.005
Toilet visits	4.253	2.421	1.352−4.336	0.003
Care of household	3.765	2.854	2.255−3.611	<0.001
Return to work	4.326	3.687	3.108−4.372	<0.001
Return to life/activities	7.085	5.748	4.731−6.984	<0.001

*Note: Logistic regression with PSF as independent factor. OR is describing the chance of independence in everyday life activities. Tested and adjusted for sex, age and depression as potential confounders*.

## Discussion

This study aimed to investigate post-stroke fatigue, its development over time, and its impact on return to work and other everyday life activities. In addition, we investigated whether PSF could predict functioning in everyday life activities one year after stroke using a large registry-based sample.

The results of the study show that PSF is a prevalent and persistent disability, as nearly half of the participants experienced PSF one year after stroke. This high prevalence of PSF is in line with the results of previous research [18,32]. Furthermore, the research findings indicate that a majority of the participants who experienced PSF were unable to resume their pre-stroke everyday life activities, including work even after one year of stroke recovery. Many stroke survivors consider work a significant and valued activity, and resuming work can serve as proof of their recovery [9]. The study confirms that the absence of PSF one year post-stroke increases the possibility of being able to return to work by almost four times. This suggests that PSF has a persistent negative impact on an individual’s ability to function and participate in everyday life, including work. Therefore, PSF must not be ignored after discharge from the hospital and during rehabilitation. However, post-stroke fatigue is an invisible impairment that may not be detected during hospital stay. Instead, it is often revealed after returning home when the person resumes activities in everyday life [10,33] or when returning to work [34].

Everyday life comprises both basic and complex activities, where basic activities such as dressing and mobility are fundamental for the performance of more complex activities [35]. In the present study, almost all participants were independent in basic activities of daily living at three months post-stroke. Difficulties with resuming activities was evidently not related to basic activities early in the recovery process. However, difficulties persisted in complex activities one year after onset of stroke, and these were significantly associated with PSF. These findings are consistent with earlier studies where PSF was found to be significantly associated with more complex activities such as shopping and preparing meals compared to basic ADL such as dressing and eating [34,36,37]. Thus, it is crucial to look beyond basic ADL and broaden the context to involve complex activities early in the rehabilitation process. Furthermore, patients who are assessed as independent in basic ADL are often discharged shortly after admission to the hospital, which leaves healthcare professionals with little time to assess and plan rehabilitation.

In the present study, one in seven participants developed PSF during the first year of recovery. This may be explained by the process of reclaiming complex activities that require the skills of organizing, planning, and flexibility in everyday life. Previous studies have yielded inconclusive results regarding the temporal aspect regarding the development of PSF [38]. Thus, in the rehabilitation process, there is a need to be aware of the development of PSF, because PSF is a subjective symptom influenced by a variety of factors, including biological, psychological, and behavioral factors [19]. Within this study the average length of hospital stay was 8.6 days, a duration that is insufficient for evaluating the development or potential occurrence of fatigue in a patient. However, periodic screening for PSF at follow-up care visits [39] is an approach that may be of use to observe whether and how PSF affects the rehabilitation process and the person’s functioning in everyday life post-stroke. However, due to a lack of knowledge concerning everyday life as a whole, healthcare professionals may not always sufficiently address complex individual needs when performing follow-ups [40]. In order to discover and clarify an individual’s multifaceted needs after stroke, the Post-stroke checklist has been suggested as a feasible tool that recognizes PSF and difficulties within the area of life after stroke three months [41] up to five years post-stroke [42]. However, it is important to ensure that the identified problems lead to appropriate interventions [43].

Based on the results of this study, the chance of returning to everyday life as before the stroke increases almost six times when not experiencing PSF one year post-stroke. However, studies have shown that patients seldom receive any information or advice about post-stroke fatigue during their hospital stay or after discharge [33,34,44] and that they are omitted to learn to manage fatigue by themselves [33]. As knowledge about life after stroke is currently limited, national guidelines in Europe do not contain any applicable recommendations regarding inter­ventions that facilitate participation and integration in society, including return to work [45]. However, there is an emerging base of knowledge regarding fatigue-management programs and interventions aimed at improving PSF that needs to be further studied [39]. Awaiting for robust evidence, healthcare professionals must recognize and acknowledge how PSF impacts complex activities in a unique individual’s life.

In accordance with previous research [21,46–48], the study showed that women experience PSF to a greater extent than men, both at the 3- and 12-month follow-ups. Sex differences have been found to persist up to seven years after stroke [21]. Differences in daily life responsibilities can be the result of women tending to take on greater responsibility for housework and childcare while employed [46]. In Sweden, women generally spend more time on unpaid household work and care for others than men. Women also experience more stress than men because they have too much to do, regardless of whether they live with somebody [49]. Further knowledge is needed regarding PSF and sex differences in relation to the ability to return to everyday activities, including work. To understand the complex symptoms of PSF both as a whole affecting everyday life and regarding sex differences, researchers should consider using a biopsychosocial perspective as a theoretical framework [22]. However, fatigue is an ambiguous term and better accepted operation definitions are required [50].

This registry-based study included a considerably large and representative study population, with high response rates at the 3- (82%) and 12-months (73%) [51,52] follow-ups post-stroke. All 72 hospitals in Sweden that admitted patients with acute stroke collected data and contributed to the Riksstroke registry. In addition, the registry is one of the few international stroke registries that collects patient-reported outcomes and patient-reported experiences. The questionnaires used at 3- and 12-months were considered valid in terms of content validity, face validity, and readability [53]. However, this study has some limitations. Currently, there is a lack of consensus regarding how to define and assess PSF [22,54] and in our study a self-reported question was used to define PSF. By assuming that the response option ‘Sometimes’ indicated normal fatigue, we attempted to distinguish between morbid and normal fatigue. Holmberg et al. [55] argued for and defined fatigue by dichotomizing the answers to the question in this way. Self-rated depressed mood was chosen as a confounder, as previous research [19–21] has shown a correlation between depression and PSF, which is consistent with our study. There is a shortage of information about the respondents’ quality of sleep, which can be considered a limitation in the study because sleep disturbances are common after stroke and have been associated with PSF [17].

## Conclusion

In summary, this study demonstrates that fatigue after stroke is prevalent and constitutes an extensive problem in managing everyday life for people of working age. Furthermore, the study identified self-reported fatigue one year after stroke as a crucial determinant of functioning in complex activities in daily life. Therefore, it is essential for rehabilitation professionals to implement routine post-discharge assessments of PSF and functioning in everyday life, with the potential to commence or restart rehabilitation measures as needed.

## Implications for practice, policy, and research

There is a need to look beyond basic ADL and consider the impact of PSF on everyday life, including work, when planning rehabilitation after stroke for working age people. Additionally, the patient’s recovery process should be followed when resuming activities in everyday life, as this can affect the development of post-stroke fatigue. Guidelines for stroke rehabilitation need to include the context of everyday life, including complex activities, such as work. Further research is needed to elucidate how PSF can be effectively managed to facilitate people’s ability to return to and maintain work and everyday activities.

## Data Availability

The released dataset can only be used for purposes that have been applied and approved by the Swedish Ethical Review Authority. The Swedish Stroke Register (Riksstroke) is not publicly available but can be made available upon reasonable application to registry managers.
