# Correction

**DOI:** 10.1080/07853890.2024.2373533

**Published:** 2024-07-01

**Article title:** The impact of post-stroke fatigue on work and other everyday life activities for the working age population – a registry-based cohort study

**Authors:** Jessica Vollertsen, Mathilda Björk, Anna-Karin Norlin and Elin Ekbladh

**Journal:** *Annals of Medicine*

**Bibliometrics:** Volume 55, Number 02, page 2269961

**DOI:** https://doi.org/10.1080/07853890.2023.2269961

When the above article published online, it contained incorrect values in Table 4. This has been corrected and republished with the accurate information. The corrected Table 4 is given below.

**Table 4.** Lack of post-stroke fatigue and prediction of positive functioning in everyday life activities one year after stroke.

Independence in activities	OR	Adjusted OR	95% CI for adjusted OR	*p*
Mobility	3.698	2.359	1.639–3.394	<0.001
Dressing	3.343	1.896	1.212–2.968	0.005
Toilet visits	4.253	2.421	1.352–4.336	0.003
Care of household	3.765	2.854	2.255–3.611	<0.001
Return to work	4.326	3.687	3.108–4.372	<0.001
Return to life/activities	7.085	5.748	4.731–6.984	<0.001

*Note*: *Logistic regression with PSF as independent factor. OR is describing the chance of independence in everyday life activities. Tested and adjusted for sex, age and depression as potential confounders*.

